# Systematic Limitations in Concentration Analysis via Anomalous Small-Angle X-ray Scattering in the Small Structure Limit

**DOI:** 10.3390/polym8030085

**Published:** 2016-03-16

**Authors:** Guenter Goerigk, Sebastian Lages, Klaus Huber

**Affiliations:** 1 Soft Matter and Functional Materials, Helmholtz-Zentrum für Materialien und Energie GmbH, Hahn-Meitner-Platz 1, D-14109 Berlin, Germany; 2 Department Chemie, Fakultät für Naturwissenschaften, Universität Paderborn, D-33098 Paderborn, Germany; klaus.huber@upb.de; 3 Max IV Laboratory, Lund University, SE-221 00 Lund, Sweden; sebastian.lages@maxlab.lu.se

**Keywords:** counterion condensation, ASAXS, RI analysis, Monte Carlo simulations, small structure limit

## Abstract

Anomalous small angle scattering measurements have been applied to diluted solutions of anionic polyacrylates decorated by specifically-interacting Pb^2+^ cations, revealing partial collapse of the polyacrylate into pearl-like subdomains with a size on the order of a few nanometers. From the pure-resonant scattering contribution of the Pb^2+^ cations, and from subsequent analysis of the resonant-invariant, the amount of Pb^2+^ cations condensed onto the polyanions with respect to the total amount of Pb^2+^ cations in the solvent was estimated. In order to scrutinize systematic limitations in the determination of the chemical concentrations of resonant scattering counterions in the collapsed phase, Monte Carlo simulations have been performed. The simulations are based on structural confinements at variable size in the range of few nanometers, which represent the collapsed subdomains in the polyanions. These confinements were gradually filled to a high degree of the volume fraction with resonant scattering counterions giving access to a resonant-invariant at a variable degree of filling. The simulations revealed in the limit of small structures a significant underestimation of the true degree of filling of the collapsed subdomains when determining chemical concentrations of Pb^2+^ cations from the resonant invariant.

## 1. Introduction

The density of charges along a polyelectrolyte chain makes the chain conformation sensitive to electrolytes. The two most efficient strategies to control changes in polyelectrolyte conformation are the screening of electrostatic interactions among charged polymer segments by adding an inert salt [1] and the neutralization of charges on the polyelectrolyte chain by adding specifically-interacting counterions [2,3]. Synthetic polyelectrolytes may, therefore, be used as simple models for biopolymers, where the role of electric charges is essential for the proper functioning of nucleic acids, numerous enzymes, and proteins.

Theoretical understanding of the mechanism underlying the impact of specifically-interacting cations has made significant progress. To give but an example, the shape of the anionic polyacrylate coils gradually changes from a coil to a compact sphere with increasing electrostatic discharging via binding of specifically-interacting alkaline earth cations, thereby passing a cascade of transition states. For these transition states cigar-like or pearl necklace structures are discussed. The latter were predicted in analogy to the Rayleigh instability of oil droplets while being electrically charged [4,5]. Beyond all doubt, the actual shape depends, in a subtle way, on the counterion concentration and interactions between solvent and chain backbone [4,5,6,7,8,9,10]. Although, the pearl necklace model has attracted much attention [11,12,13,14,15], a quantitative analysis of the collapsed domains is still lacking.

Some progress in this direction could be achieved with anomalous small-angle X-ray scattering (ASAXS). ASAXS enables the structural characterization of the counterion distribution around the macroions and a quantitative estimation of the amount of condensed counterions by tuning the energy in the vicinity of the absorption edge of the counter ion in question. The distribution of the counterions is not accessible by conventional SAXS measurements, because the scattering contributions of the counterions and the macroions superimpose and cannot be distinguished. The first ASAXS experiments of counterion distributions were reported by Stuhrmann [16] and more recent results are published in [15,17,18,19,20,21,22,23,24,25]. In several of those studies sodium polyacrylate (NaPA) chains were subjected to the addition of two different divalent cations [M^2+^] with M^2+^ equal to Sr^2+^ or Pb^2+^ as two examples for the impact of specifically interacting cations on the conformation and solubility of NaPA chains. The specifically interacting M^2+^ cations form complex bonds with the anionic carboxylate residues, located on every other carbon atom of the polymer backbone. Formation of complex bonds between the anionic groups and the M^2+^ cations neutralizes electric charges and thereby changes the nature of the respective chain segments. As a consequence, the solubility of the polyelectrolyte is lowered which leads to a significant coil shrinking and eventually causes a precipitation of the respective M^2+^-salt [2,3,14,15]. Noteworthy, the studies where NaPA chains have been subjected to the addition of Sr^2+^ or Pb^2+^ revealed a degree of decoration of the NaPA chains, which remained below the expected value with a larger discrepancy in case of Pb^2+^-PA [23,24]. Whereas, in the case of the Sr^2+^ cationss ASAXS suggested a percentage of neutralization of the COO^−^ groups of 20%–33% compared to the anticipated value of 50% [23], the apparent degree of neutralization suggested by ASAXS in case of Pb^2+^ was only 5%–10% compared to an expected value close to 30% [24].

In order to shed further light on the validity of quantitative ASAXS, in general, and on this discrepancy, in particular, a combined SANS and ASAXS investigation has been performed on the behavior of NaPA chains, subjected to the addition of divalent Pb^2+^ cations as the specifically-interacting cation. As has just been mentioned, the Pb^2+^ cations showed a particularly large discrepancy between anticipated amount of Pb^2+^ cations and the respective amount recovered by ASAXS. In the present study, D_2_O has been used as solvent in order to enable a combined small angle neutron scattering (SANS) and SAXS study. SANS at the same sample where ASAXS is carried out shall provide an independent access to the shape of the collapsing NaPA coil with a focus on the organic matrix, hosting the lead cations. Accordingly, we expect to get a detailed picture on the size and shape of the “confinement”, which are formed by the collapsing NaPA coils and which serve as containers for the condensing Pb^2+^ cations. The amount of Pb^2+^ cations in solution is selected to create samples close to the precipitation threshold of Pb^2+^-PA, as under such conditions intermediates with a significant degree of collapse are achieved. Deduction of the amount of Pb^2+^ in the collapsed phase is inferred from ASAXS by a precise analysis of what has been introduced as the resonant-invariant (RI) in a former publication [23]. The concentrations of Pb^2+^ ions in the condensed phase deduced accordingly from the ASAXS measurements are compared to Monte Carlo simulations.

### Anomalous Small-Angle X-Ray Scattering

The remarkable possibilities of the ASAXS techniques are based on the energy dependence of the atomic scattering factors giving selective access to the specific SAXS contributions of nano-scaled phases, which are built up by different chemical constituents for instance clouds of Pb^2+^-counterions, which surround negatively-charged polyacrylate chains. In general, the atomic scattering factors are energy dependent complex quantities:

(1) $$f_{Z}(E)=f_{0,Z}+f_{Z}^{\prime }(E)+if_{Z}^{\prime \prime }(E)$$

where *Z* represents the atomic number. When performing ASAXS measurements on Pb^2+^counterions in the vicinity of the L_III_-absorption edge of Pb at 13,035 eV the scattering amplitude is:

(2) $$A(\vec{q},E)=\int_{V}\Delta \rho_{M}(\vec{r})\cdot \text{exp}(i\vec{q}\vec{r})d^{3}r+\int_{V}\Delta \rho_{\text{Pb}}(\vec{r},E)\cdot \text{exp}(i\vec{q}\vec{r})d^{3}r$$

where *q* is the magnitude of the scattering vector [=(4π/λ)sinΘ], ${\Delta \rho }_{M},{\Delta \rho }_{\text{Pb}}$ 2Θ is the scattering angle, λ the X-ray wavelength and *V* is the irradiated sample volume. ${\Delta \rho }_{M},{\Delta \rho }_{\text{Pb}}$ are the differences of electron densities of the non-resonant scattering monomer units of the polyacrylates and the resonant scattering Pb^2+^counterions respectively:

(3) $$\begin{array}{l} \Delta \rho_{M}(\vec{r})=\Delta f_{M}\cdot u(\vec{r})=(f_{M}-\rho_{m}V_{M})\cdot u(\vec{r})\\ \Delta \rho_{\text{Pb}}(\vec{r},E)=\Delta f_{\text{Pb}}(E)\cdot v(\vec{r})=((f_{0,\text{Pb}}-\rho_{m}V_{\text{Pb}})+f_{\text{Pb}}^{\prime }(E)+if_{\text{Pb}}^{\prime \prime }(E))\cdot v(\vec{r}) \end{array}$$

where $\rho_{m}$ is the electron density of the entire solution. The volume $V_{M}$ represents the volume of the non-resonant scattering monomer unit and $V_{\text{Pb}}$ corresponds to the volume of the resonant scattering Pb^2+^ ion. The functions $u(\vec{r}),v(\vec{r})$ are the number densities of monomer units, specified in more detail below, and of Pb^2+^ ions representing the respective spatial distribution in the sample. The monomeric scattering factor, $f_{M}(E)\approx const$, is nearly energy independent, while the atomic scattering factor, $f_{\text{Pb}}(E)=f_{0,\text{Pb}}+f_{\text{Pb}}^{\prime }(E)+if_{\text{Pb}}^{\prime \prime }(E)$, shows a strong variation with the energy in the vicinity of the L_III_-absorption edge of the resonant scattering Pb due to the so-called anomalous dispersion corrections $f_{\text{Pb}}^{\prime }(E),f_{\text{Pb}}^{\prime \prime }(E)$. Calculating the scattering intensity $I(\vec{q},E)={\left|A(\vec{q},E)\right|}^{2}=A(\vec{q},E)\cdot A^{*}(\vec{q},E)$ by means of Equations (2)–(3), and averaging over all orientations, yields the sum of three basic scattering contributions [16]:

(4) $$I(q,E)=S_{M}(q)+S_{\text{MPb}}(q,E)+S_{\text{Pb}}(q,E)$$

with:

(5) $$\begin{array}{cc} S_{M}(q)= & \Delta f_{M}^{2}{\left|A_{M}(q)\right|}^{2}\\ S_{\text{MPb}}(q,E)= & 2\Delta f_{M}\cdot (f_{0,\text{Pb}}-\rho_{m}V_{\text{Pb}}+f_{\text{Pb}}^{\prime }(E))\cdot \text{Re}(A_{M}(q)A_{\text{Pb}}(q))\\ S_{\text{Pb}}(q,E)= & [{\left(f_{0,\text{Pb}}-\rho_{m}V_{\text{Pb}}+f_{\text{Pb}}^{\prime }(E)\right)}^{2}+{f_{\text{Pb}}^{\prime \prime }}^{2}(E)]{\left|A_{\text{Pb}}(q)\right|}^{2} \end{array}$$

and:

(6) $$\begin{array}{cc} {\left|A_{M}(q)\right|}^{2}= & \iint_{V_{P}}u(\vec{r})u(\vec{r^{\prime }})\frac{\text{sin}(q\left|\vec{r}-\vec{r^{\prime }}\right|)}{q\left|\vec{r}-\vec{r^{\prime }}\right|}d^{3}rd^{3}r^{\prime }\\ \text{Re}(A_{M}(q)A_{\text{Pb}}(q))= & \iint_{V_{P}}u(\vec{r})v(\vec{r^{\prime }})\frac{\text{sin}(q\left|\vec{r}-\vec{r^{\prime }}\right|)}{q\left|\vec{r}-\vec{r^{\prime }}\right|}d^{3}rd^{3}r^{\prime }\\ {\left|A_{\text{Pb}}(q)\right|}^{2}= & \iint_{V_{P}}v(\vec{r})v(\vec{r^{\prime }})\frac{\text{sin}(q\left|\vec{r}-\vec{r^{\prime }}\right|)}{q\left|\vec{r}-\vec{r^{\prime }}\right|}d^{3}rd^{3}r^{\prime } \end{array}$$

Equation (5) gives the non-resonant scattering, $S_{M}(q)$, the cross-term or mixed-resonant scattering, $S_{\text{MPb}}(q,E)$, originating from the superposition of the scattering amplitudes of the non-resonant polyacrylates, and the resonant scattering Pb^2+^ ions and finally, $S_{\text{Pb}}(q,E)$, which contains only the scattering contributions of the resonant scattering Pb^2+^ ions. ${\left|A_{M}(q)\right|}^{2}$ and ${\left|A_{\text{Pb}}(q)\right|}^{2}$ in Equation (6) represent the squared scattering amplitudes averaged over all orientations (Debye), which depend only on the number density distributions of the monomers and the Pb^2+^-counterions, respectively, thereby liberated from their contrast with respect to the solvent. In analogy *Re(A*_M_*(q)A*_Pb_*(q))* denotes the (averaged) superposition of the scattering amplitudes of the monomer and the Pb^2+^-counterions.

When solving the vector equation constituted by ASAXS measurements at a minimum of three energies by the Gaussian elimination procedure, the form factor ${\left|A_{\text{Pb}}(q)\right|}^{2}$ can be determined in analytical form [15,23,24,25,26,27]:

(7) $${\left|A_{\text{Pb}}(q)\right|}^{2}=\left[\frac{I(q,E_{1})-I(q,E_{2})}{f_{\text{Pb}}^{\prime }(E_{1})-f_{\text{Pb}}^{\prime }(E_{2})}-\frac{I(q,E_{1})-I(q,E_{3})}{f_{\text{Pb}}^{\prime }(E_{1})-f_{\text{Pb}}^{\prime }(E_{3})}\right]\cdot \frac{1}{F(E_{1},E_{2},E_{3})}$$

where *F(E*_1_,*E*_2_,*E_3_)* represents a normalization factor composed of the anomalous dispersion corrections at the respective three energies *E*_1_, *E*_2_, *E*_3_. A comprehensive description of the mathematical details can be found in [28].

${\left|A_{\text{Pb}}(q)\right|}^{2}$ is the Fourier transform of the pair correlation function of the resonant scattering Pb^2+^-counterions. Thus, Equation (7) provides direct access to the small-angle scattering of Pb^2+^ ion assemblies and, along with it, structural information on the distribution of the Pb^2+^-counterions. More generally spoken, Equation (7) provides a method, which gives access to the pure-resonant scattering contribution of the Pb^2+^-counterions by measuring the small-angle scattering at only three suitable energies.

In addition to the structural information, which can be obtained from ${\left|A_{\text{Pb}}(q)\right|}^{2}$, important quantitative information related to the amount of inhomogeneously-distributed Pb^2+^ ions can be deduced from the integral [23]:

(8) $$Q_{\text{Pb}}(E)/{\left|\Delta f_{\text{Pb}}(E)\right|}^{2}=\int_{Q}{\left|A_{\text{Pb}}(q)\right|}^{2}d^{3}q$$

In analogy to the so-called invariant [29,30], we will call $Q_{\text{Pb}}(E)/{\left|\Delta f_{\text{Pb}}(E)\right|}^{2}$ the resonant-invariant (RI) of the inhomogeneously-distributed resonant scattering Pb^2+^ ions. The RI, as defined in Equation (8), is related to the number density of inhomogeneously-distributed Pb^2+^ ions, $\bar{v_{\text{Pb}}}$, as was outlined in detail in [23,27,31,32]:

(9) $$\begin{array}{r} \bar{v_{\text{Pb}}}=\frac{1}{2V_{\text{Pb}}}\pm \sqrt{\frac{1}{4V_{\text{Pb}}^{2}}-\frac{1}{{\left(2\pi \right)}^{3}r_{0}^{2}}\int_{0}^{q_{SAXS}}{\left|A_{\text{Pb}}(q)\right|}^{2}d^{3}q} \end{array}$$

where *r_0_* is the classical electron radius and the volume of the Pb^2+^ ions $V_{\text{Pb}}=4\pi R_{\text{Pb}}^{3}/3$ is estimated with the ion radius *R*_Pb_. Due to the integral in the square root, Equation (9) provides the quantitative analysis of concentration fluctuations of the chemical species Pb^2+^, independent of its spatial distribution. As indicated by the upper integration limit in Equation (9) the integral is extended up to *q*_SAXS_, which is predefined by the setup of the SAXS experiment. In order to analyze the systematic limitations introduced by *q*_SAXS_ when determining the chemical concentrations Monte-Carlo calculations (MC) have been performed. The MC calculations were designed to simulate the scattering of Pb^2+^ ions confined in a small volume predetermined by the shrinking polyacrylate coils and suggested experimentally by our SAXS and SANS analyses of the respective samples (see below). Specifically, we have adopted cube-like containers to model the collapsed subdomains in the shrinking Pb^2+^-PA coils. As a result, simulated scattering curves are received, which are processed with Equation (9) in an analogous way as our experimental ASAXS curves. Up to degrees of filling (volume fractions) of 50% only one of the two solutions in Equation (9) is significant *i.e.* the one with the negative sign. Beyond 50% the calculated values estimate only the lower limit, which is obtained by extrapolation of Equation (9) to higher degrees of filling by using the negative sign. The positive sign over estimates strongly the concentrations and is not further considered.

In order to scrutinize to which extent such application of Equation (9) recovers the true amount of Pb^2+^ ions in the confinements this true value, *i.e.* the true invariant has to be known. The true invariant is given by all photons scattered in all directions in space (and not only recorded by the limited solid angle of the detector). This true invariant is accessible for our present simulations and calculated as follows. The scattering deduced from the MC calculations is extended over the “entire reciprocal space”, defined by the wavelength of the (A)SAXS experiment under consideration and by the maximum scattering angle (2θ = 180°). In our present experiment the wavelength is predefined by the energy of the L_III_-absorption edge of Pb (13,035 eV) to λ = 0.091 nm which results in a maximum value of *q*_max_ = 136 nm^−1^ for an integration which is the “entire reciprocal space”. Integration of the scattering cross-section accordingly provides the true invariant, *Q*_max_, which is directly related to the number *N* of Pb ions per cubic-like confinement:

(10) $$\begin{array}{ll} Q_{\text{max}} & =\frac{4\pi }{3}q_{\text{max}}^{3}\left\{N+2\sum_{i=1,j>i}^{N}3\frac{\left[\text{sin}(q_{\text{max}}\left|{\vec{r}}_{i}-{\vec{r}}_{j}\right|)-q_{\text{max}}\left|{\vec{r}}_{i}-{\vec{r}}_{j}\right|\text{cos}(q_{\text{max}}\left|{\vec{r}}_{i}-{\vec{r}}_{j}\right|)\right]}{q_{\text{max}}^{3}{\left|{\vec{r}}_{i}-{\vec{r}}_{j}\right|}^{3}}\right\}\\ & -{\left(2\pi \right)}^{3}N{\bar{v}}_{\text{Pb}} \end{array}$$

with ${\bar{v}}_{\text{Pb}}=N/V_{P}$ and *V*_P_ the volume of the confinement (for details see Supplementary Materials). (A)SAXS experiments are restricted to a limited *q*-range *q*_SAXS_ and by no means cover the above introduced “entire reciprocal space”. Thus, Equation (10) cannot be used for the calculation of chemical concentrations because it is valid only if the integration of the scattering cross sections is extended over the entire q-space limited by *q*_max_. This q-space is inevitably much larger than that covered by *q*_SAXS_. However, the chemical concentrations of inhomogeneously-distributed Pb^2+^ ions can be estimated from scattering curves in the limited SAXS regime by means of Equation (9) which is based on Porod’s theory [30]. Porod assumed, that only sample inhomogeneities established by deviations from the average scattering length density contribute to the scattering in the SAXS regime. A detailed derivation of Equation (9) was outlined in [23].

In case of the ASAXS experiments the degree ε to which the amount of Pb^2+^ ions in the confinement is recovered by Equation (9) in the limited *q*-range of 0 nm^−1^ ≤ *q* ≤ *q*_SAXS_ can be deduced from the ratio of ${\bar{v}}_{\text{Pb}}$ given in Equation (9) and N as defined in Equation (10) with the 2nd term in the bracket vanishing while integrating up to *q*_max_:

(11) $$\varepsilon =\frac{{\bar{v}}_{Pb}V_{P}}{N}=\frac{{\bar{v}}_{Pb}V_{P}\frac{4\pi }{3}q_{\text{max}}^{3}}{Q_{\text{max}}+{\left(2\pi \right)}^{3}\frac{N^{2}}{V_{P}}}$$

For Kratky curves from MC simulations, the ratio ε can be calculated alternatively by numerically integrating these Kratky curves up to the limit of *q*_max_.

## 2. Materials and Methods

NaNO_3_ and Pb(NO_3_)_2_ of analytical grade were purchased from Fluka, (Buchs, Switzerland), NaOD (40% *w*/*w* in D_2_O) was purchased from Sigma-Aldrich (Taufkirchen, Germany) and the sodium polyacrylate standard P585 was purchased from Polymer Standards Service (Mainz, Germany). D_2_O from Deutero GmbH (Kastellaun, Germany) was used without further purification.

The pH value of a solution of 0.1 M NaNO_3_ in D_2_O was set to 9 using 0.1 M NaOD in D_2_O. A stock solution of the NaPA standard was prepared in 0.1 M NaNO_3_ as solvent and gently mixed for three days. Another stock solution was prepared containing 5 mM Pb(NO_3_)_2_ and 90 mM NaNO_3_ in D_2_O. Both stock solutions were used to prepare the samples FRM01 and FRM02 for the small angle scattering experiments by mixing appropriate amounts of the 0.1 M NaNO_3_ solution, the NaPA stock solution, and the stock solution containing the lead ions. The composition of the two samples can be taken from Table 1.

The ASAXS measurements were carried out at the beam line B1 (former JUSIFA) at HASYLAB, DESY Hamburg [33,34] using a double-crystal (Si-311) monochromator with an energy resolution of about $\Delta E/E\approx 3\times 10^{-5}$ covering a *q*-range between 0.06 and 2.5 nm^−1^ at three energies in the vicinity of the L_III_-absorption edge of Pb at 13035 eV. Table 2 provides the anomalous dispersion corrections for Pb at the three energies used here based on the calculations of Cromer and Liberman [35,36]. The scattering patterns have been detected with a 300 k Pilatus detector (DECTRIS, Baden-Daettwil, Switzerland). All scattering patterns have been normalized and corrected for background and transmission. The scattering of the solvent was subtracted from the sample scattering. Transmission measurements were performed with a precision better than 10^−4^ using a special (windowless) photodiode (Hamamatsu S2387-1010N, Hamamatsu, Japan). The scattering intensity was calibrated into an absolute macroscopic scattering cross section in units of cross-section per unit volume [cm^2^/cm^3^] = [cm^−1^] by use of the JUSIFA reference (glassy carbon) standards. The ASAXS sequences covering three energies followed the JUSIFA standard procedures *i.e.* repeating the whole sequence several times with subsequent averaging [27]. The two samples selected for ASAXS as well as the solvent were filled into capillaries from Hilgenberg GmbH (Malsfeld, Germany). The capillaries are made of borosilicate glass with an inner diameter of 2 mm and a wall thickness of 0.05 mm. The capillaries were closed with a pipette plug fixed by two-component quick setting adhesive.

The SANS measurements have been performed at the Jülich Center for Neutron Science (JCNS) with the beam line KWS-2 at the FRM II (Forschungsreaktor München of the Technische Universität München, Garching, Germany) [37]. The samples were filled in standard Helma cells with a thickness of 2 mm and have been exposed to a neutron beam of wavelength 0.7 nm at sample detector distances of 2, 8, and 20 m. Along with the sample solvent measurements have been performed using 0.1 M NaNO_3_ in D_2_O.

## 3. Results

**Figure 1:**
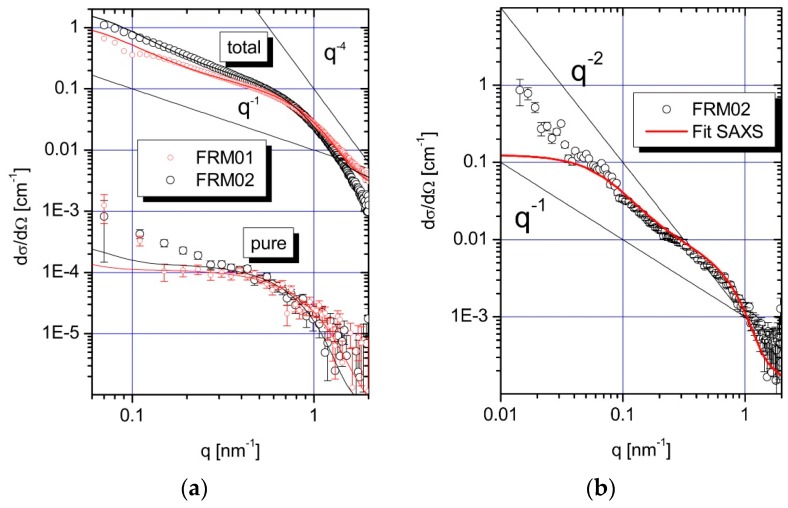
(**a**) SAXS curves of samples FRM01 and FRM02 measured at three different X-ray energies in the vicinity of the LIII-absorption edge of Pb at 13,035 eV. The curves with the label “total” represent the total scattering, while the curves with label “pure” represent the form factor of the pure-resonant contribution. Note that the separated form factor of the pure-resonant scattering contribution is more than three orders of magnitude smaller with a cross-section down towards 10^–5^ cm^−1^ (!) when compared to the total scattering (factor 2000); (**b**) SANS curve of the sample FRM02. The solid lines running through the symbols of the scattering curves are fitted their respective scaled model functions representing the “freely jointed chain pearl necklace” FJC-PN model (see text).

Figure 1 compares the scattering curves obtained from SAXS for samples FRM01 and FRM02 (a) with SANS experiments performed with sample FRM02 (b). All curves have in common that they indicate a shallow shoulder in the regime of 0.5 nm^−1^ < *q* < 1 nm^−1^, which suggests the occurrence of dense domains in the order of magnitude of a few nanometers. In agreement with our preceding experiments on the same system [24], the present data confirms the existence of partially collapsed intermediate states of the polyacrylate chains. The collapse is induced by the binding of Pb^2+^ ions, which generates densely packed pearl-like subdomains in the chain, interconnected by string-like non-collapsed chain segments. The total scattering curves from SAXS show slight differences for the two samples. The scattering curve of FRM02, which is closer to the phase boundary, reveals a larger scattering cross-section in the low *q*-regime and, in parallel, a distinct *q*^−4^-behavior (Porod) at larger *q*-values indicating that the formation of collapsed phases is more evolved when compared to FRM01.

In order to analyze the samples in terms of structurally characteristic parameters more quantitatively, we applied the same model as introduced in [24]. The model is a chain of m freely-jointed rod-like monomers each of bond length *l*, which includes *n* equally-spaced spheres of radius R, with any two neighboring spheres separated by *m*/(*n*−1) rod-like monomers. The model is, hereafter, denoted as a freely-jointed chain pearl necklace (FJC-PN). The spheres represent the collapsed subdomains and the freely jointed chain segments the interconnecting strings. Potential polydispersity of the pearls is accounted for by a Schulz–Zimm distribution of the sphere volume (see model-2 in [24]). The model fits qualitatively reproduce the total SAXS curves and are particularly satisfactory in the regime of 0.2 nm^−1^ < *q* < 2 nm^−1^ From the fit curves radii between 2.6 and 3.4 nm for the collapsed domains have been deduced with less than five pearls correlating in a volume of about 30 nm size, offered by the partially collapsed polyacrylate chain.

Figure 1b summarizes the results obtained from SANS measurements of FRM02 only because the data quality of FRM01 is not sufficient for a further interpretation. For comparison we have plotted the fit curve of the FJC-PN model obtained from SAXS via scaling into the SANS curve of FRM02 (solid line). Interestingly the FJC-PN model reproduces the SANS curve over one order in magnitude of the q-range between 0.1 and 2 nm^−1^. An explanation will be given in the discussion.

Figure 1a also compiles the results of the SAXS curves measured at three different X-ray energies in the vicinity of the L_III_-absorption edge of Pb at 13035 eV of NaPA in D_2_O. From Equation (7) the form factor of the pure-resonant scattering was calculated (symbols with the label “pure”). The solid lines running through the symbols represent the self-term of the pearls from the FJC-PN-model scaled to the form factor of the pure-resonant scattering. This nicely confirms that the scattering of the Pb^2+^ ions is perfectly reproduced by the pearl-like collapsed subdomains.

**Figure 2:**
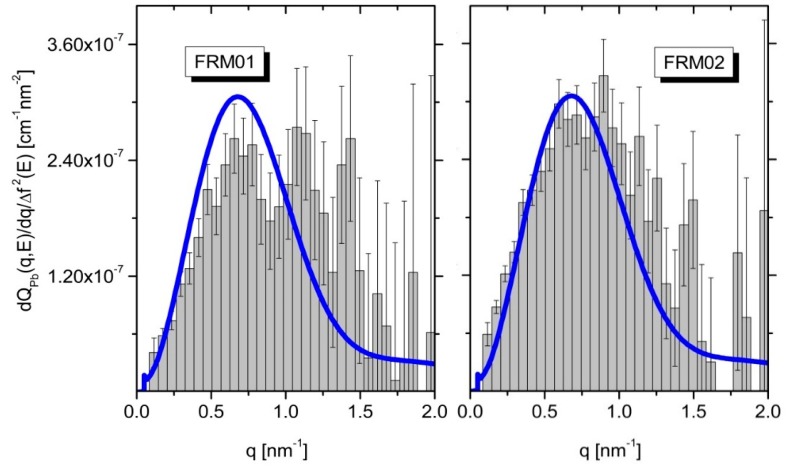
The resonant invariant of the Pb counterions. From the integral the Pbconcentrations in the condensed phase is deduced. The blue lines represent the FJC-PN model (see text).

**Figure 3:**
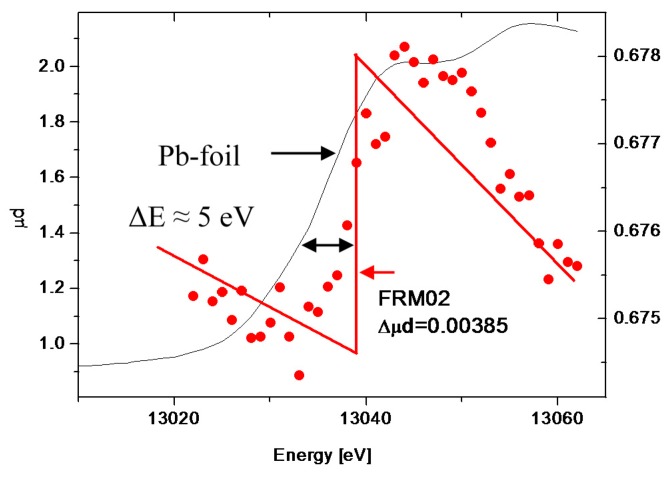
XANES spectrum of FRM02 measured at the Pb L_III_-edge at 13,035 eV. The solid (black) line represents the XANES spectrum of a Pb-metal foil (*y*-axis on the left). The FRM02 spectrum reveals a chemical shift of about 5 eV. Δμ*d* is the difference of the absorption coefficient times the sample thickness and represents the Pb-L_III_-edge of the sample taken from the *y*-axis on the right.

Figure 2 depicts the RI of the Pb^2+^counterions for the two samples. From the integrals of the ASAXS curves (grey area in Figure 2) the Pb^2+^concentrations localized in the condensed phases have been calculated and compared with the Pb^2+^concentrations obtained from the XANES measurements (Figure 3) of the two samples. The results are summarized in Table 1. The Pb^2+^content obtained from the XANES measurements is in good agreement with the value known from the sample preparation, while the comparison with the chemical concentrations obtained from the RI analysis tells us that only about 10%–14% of the Pb^2+^counterions are detected in the collapsed domains (see discussion).

## 4. Discussion

Since neutron scattering is mainly sensitive to the organic component the SANS curves represent the superposition of the scattering originating from the NaPA chains and the incorporated Pb^2+^ ions. Contributions from the PA chains is larger than from the Pb^2+^ ions because, at best, 40% of the anionic monomers are directly bound to divalent Pb^2+^ ions, while 60% of the anionic residues are not. In case of the SAXS curves the situation is different. Here, the contrast of the heavy Pb^2+^ ions is large with respect to the solvent, and the contrast of the organic PA chains is weak. Hence, the SAXS curves are dominated by the scattering of the Pb-containing PA segments. Having available a SAXS and a SANS curve from the same sample FRM02, we are able to draw significant conclusions with respect to the internal structure of the Pb–PA complex. The FJC-PN model successfully reproduces the SAXS curve of this sample in the entire q-regime available. Strikingly, the same model curve reproduces equally well the respective SANS curve in the entire regime of 0.07 < q < 2 nm^−1^ accessible to the SAXS experiment. This coincidence demonstrates, that the SAXS curve runs parallel with the SANS curve in this q space, thus proving that the structural features of the PA chain is essentially identical with the spatial distribution of the Pb^2+^ ions bound to this chain in the corresponding size window. Clearly, the structure of the collapsing NaPA chain is reproduced by the bound Pb^2+^ ions.

We now turn to the quantitative analysis of the ASAXS experiments *i.e.* the analysis of the resonant invariant in order to determine the amount of Pb^2+^ ions captured in the suspected collapsed subdomains (pearls). We emphasize that no model functions are required for the quantitative analysis of the resonant invariant. The grey areas in Figure 2 simply represent the chemical concentrations of the Pb^2+^ ions located in the polymer chains and, therein, predominantly in the collapsed domains. Although the self-term of the pearls from the FJC-PN model suggests that it essentially reproduces the grey areas, the Pb^2+^-contents obtained from the XANES measurements (Figure 3) show strong discrepancies from the chemical concentrations obtained with the ASAXS-based RI-analysis. The ASAXS based RI analysis indicates that only between 11% (FRM01) and 14% (FRM02) of the Pb^2+^counterions are detected as being in the collapsed subdomains (last column in Table 1). This can be attributed to two different origins, respectively: (i) an incomplete q-range is covered by the present ASAXS experiments, to which the integration of the Kratky-plot is limited; and (ii) there are in fact much less Pb^2+^ cations absorbed into the collapsed domains than are expected from a complete neutralization of the COO^−^ residues of the polyacrylate chains by Pb^2+^ cations and suggested accordingly by the phase diagram. It is important to note that the concentration of Pb^2+^ in both samples was only slightly sub-stoichiometric with respect to the amount of negatively-charged COO^−^ residues provided by the polyelectrolyte chains.

In order to better judge these two alternative explanations, Monte Carlo (MC) simulations have been performed providing two states of such a counterion condensation induced collapse; one relevant for SAXS and one relevant for ASAXS. The MC simulations are performed under the assumption that the only scattering entities are Pb^2+^ ions, which is correct in the case of ASAXS. In the case of SAXS this assumption does not hold, but shall still be highly instructive if used as a working hypothesis to highlight certain crucial aspects.

Simulations have been performed for confinements with sizes based on the structural information obtained from the FJC-PN model. At this stage we like to remind the reader, that these confinements represent the pearls (partially-collapsed subdomains). In detail the scattering curves of Pb^2+^ ions collected in cubic volumes of different sizes (1, 2, 4, and 6.5 nm) were simulated in the q-range between 0.1 and 136 nm^−1^. As was outlined before the value 136 nm^−1^ is the largest *q*-value, which can be achieved with a wavelength of 0.091 nm in the energy range of the L_III_-absorption edge of Pb. Different amounts of Pb^2+^ ions have been allocated inside the cubic confinement, starting with a single ion, up to numbers of Pb^2+^ ions close to the volume fraction of dense spherical packing *i.e.* 0.74. The distances between the Pb^2+^ ions were randomly chosen, but the overlap of volumes of different Pb ions was excluded or, in other words, the ions are assumed to follow the behavior of a real gas. From the random ion-ion distances the structure factor resulting from the complete assembly was calculated, thus establishing the form factor of a cubic confinement. In order to address SAXS and ASAXS aspects of the present MC simulation, two form factors have been used for the simulation of the Pb^2+^ ions collected by the polyacrylate chains. Whereas, for the simulations of SAXS measurements the form factor of Pb^2+^ ions was modeled with a sphere with a radius of 0.119 nm, the δ-function was employed as the form factor of Pb^2+^ ions for the simulation of ASAXS measurements. The latter is justified, because in the energy range of resonant scattering the atomic form factor of Pb ions represent point-like scattering centers to good approximation.

**Figure 4:**
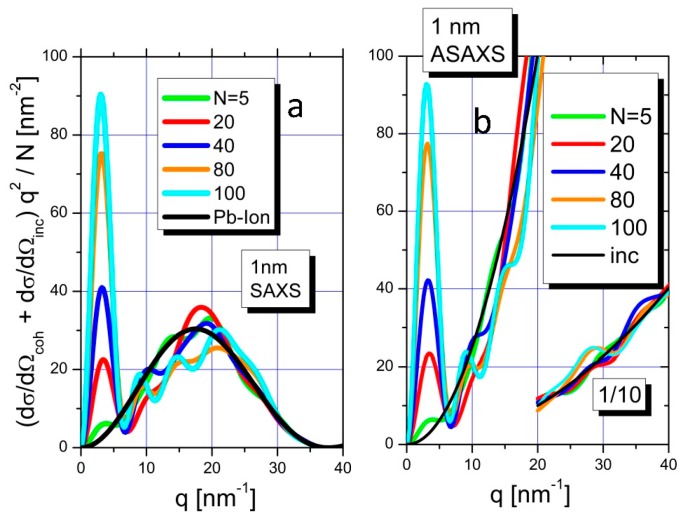
Kratky plot of scattering curves calculated from scattering curves of Pb^2+^ ions confined in a cube of 1 nm side length by Monte Carlo simulation under the condition of a standard SAXS experiment (**a**). For the Pb^2+^ ions a spherical form factor with a radius of 0.119 nm was assumed. The structure factor of 5, 20, 40, 80, and 100 Pb ions with randomly-distributed distances inside a real gas was simulated. The curves are normalized to N. The same simulations have been repeated for the real gas under the condition of ASAXS measurements using point-like scattering centers. For *q*-values beyond 20 nm^−1^ the simulated curves are divided by 10 in order to better visualize (**b**).

Figure 4a depicts the Kratky plot of the simulated scattering curves for SAXS measurements together with the Kratky plot of the spherical form factor of an isolated Pb^2+^ ion. Simulated curves include normalized Kratky plots of 5, 20, 40, 80, and 100 Pb^2+^ ions in a 1 nm confinement. Normalization was performed with the number of ions per confinement. The amount of 100 Pb^2+^ ions as the largest number of ions per confinement corresponds to a volume fraction of about 0.7 of the Pb ions in the 1 nm confinement.

The integrals of the Kratky-plots (area below curves) are directly related to the chemical concentration of Pb^2+^ ions in the simulated confinements via employment of Equation (9). As can be seen from the black curve of Figure 4a most of the integral of the form factor of an isolated Pb^2+^ ion is located at q-values beyond 10 nm^−1^ *i.e.* beyond the *q*-range of the SAXS resolution, which in the present experiment was terminated at 2 nm^−1^. The situation changes, when Pb^2+^ ions are successively filled into the confinement with 5, 20, 40, 80, or 100 Pb^2+^ ions per confinement respectively. The resulting scattering contributions in the *q*-range below 10 nm^−1^ (the SAXS range) becomes increasingly significant and finally dominant, when compared to the overall integral. Along with the increasing fill rate, the scattering curve gradually approaches the shape of the form factor of a cube.

A completely different result is established by the simulations of the ASAXS measurements. Now point-like scattering centers, instead of spherical form factors, for the scattering of the collected ions are used. Figure 4b shows the superposition of the incoherent scattering of the point-like scatterers (*i.e*., the scattering of the uncorrelated Pb^2+^ ions) with the coherent scattering originating from the Pb^2+^ ions in the confinement. The coherent scattering oscillates around the incoherent scattering when approaching larger *q*-values. The curves in the regime of *q* < 10nm^−1^ are dominated by the coherent scattering and show strong increasing scattering intensity with increasing number of Pb ions while at larger *q*-values oscillations around the parabolic increasing scattering occur. From a comparison of Figure 4a with Figure 4b it is evident, that ASAXS measurements show a significant difference of the intensity distribution in the range of 10 < *q* < 40 nm^−1^ due to the different form factors addressed by SAXS and ASAXS respectively, leading to a different degree of completeness of the integral when Equation (9) is used for the estimation of chemical concentrations.

Equation (10) was used to prove consistency of the MC simulations. In fact, Equation (10) yields the same invariant and, along with it, the full number N of scattering Pb^2+^ ions as the numerical integration of the simulated ASAXS curves does if extended over the entire *q*-regime of 0 nm^−1^ ≤ *q* ≤ *q*_max_ with *q*_max_ = 136 nm^−1^. In a truncated range of q of 0 ≤ *q* ≤ 2 nm^−1^ or of 0 ≤ *q* ≤ 10 nm^−1^ the resulting invariant contribution recovers a certain number of Pb^2+^ ions N_R_ which offers insight into the extent of completeness of the respective estimate as *N*_R_/*N* = ε given in Equation (11).

**Figure 5:**
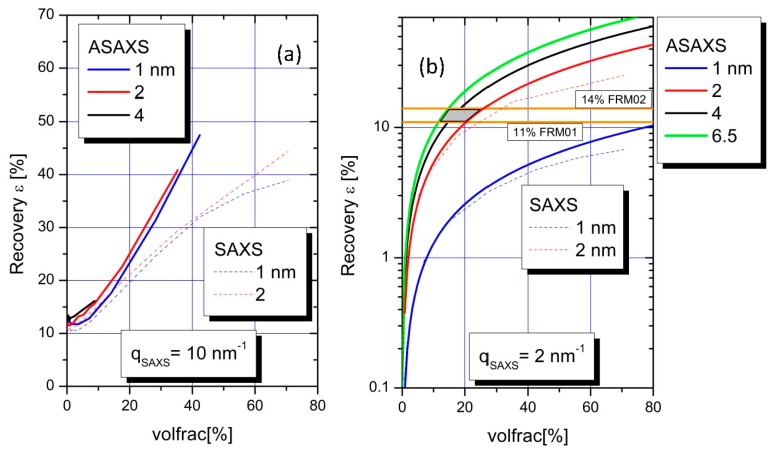
Two integrals of normalized ASAXS and SAXS Kratky plots have been calculated for four different cubic confinements (1, 2, 4, and 6.5 nm) under the condition of a standard small-angle scattering experiment over the two integration intervals 0< *q* < 10 nm^−1^ (**a**) and 0< *q* < 2 nm^−1^ (**b**). The grey parallelogram represents the area of small confinements with low filling degrees compatible with results from the RI-analysis and XANES (see text).

Figure 5 summarizes, quantitatively, the results from the MC simulations with four different cubic confinements with edge lengths of 1, 2, 4, and 6.5 nm respectively. The latter three length values have been chosen due to the results deduced from the FJC-PN model suggesting pearls with radii between 2.6 and 3.4 nm. On the y-axis the degree of recovery is plotted, which is calculated by employing Equation (11) with the integral of Equation (9) calculated for two different integration ranges of the Kratky plot: (i) from 0 to 10 nm^−1^ (Figure 5a), and (ii) from 0 to 2 nm^−1^ (Figure 5b). These integral fractions entering Equation (11) are plotted *versus* the volume fraction of the cubic confinement, which is covered by the Pb^2+^ ions up to values close to the value for hexagonal close-packing of spheres (0.74).

The RI from ASAXS achieved via integrating up to 10 nm^−1^ are indicated as the colored solid lines in Figure 5a for the different confinements (blue = 1 nm, red = 2 nm, black = 4 nm). When approaching volume fraction of 35% in the cubic confinements 41% of the scattering intensity is recovered in the case of 2 nm sized cubes, while the degree of completeness is 48% for 1 nm size for a volume fraction of 42%. Due to computer limitations the simulation was not performed for this limit in the 4 nm confinement, but it can be expected that the values converge for the larger degrees of filling. This also applies for larger confinements.

Strongly different results were found for the extended integration range in the case of SAXS measurements. The degree of recovery (completeness of the integral) is smaller in the extended *q*-range when comparing SAXS to ASAXS. This increased discrepancy can be attributed to the influence of the form factor of spatially extended ions.

For the discussion of the results calculated for the limited *q*-range up to 2 nm^−1^ in accordance with our present ASAXS experiment we switch to a logarithmic scale on the y-axis (Figure 5b) in order to visualize with a better resolution. The results are represented by the colored solid lines for the different confinements (blue = 1 nm, red = 2 nm, black = 4 nm, and green = 6.5 nm). In a linear plot, the curves are straight lines. Thus, the linear functions can be extrapolated to values of spherical dense packing for the large confinements (4 and 6.5 nm). When approaching close packing of the cubic confinements nearly 70% of the scattering intensity is recovered in the case of 6.5 nm sized pearls while the degree of completeness is only 9% for 1 nm size. Significant differences appear for the trends in recovered scattering curves of ASAXS and SAXS measurements in this *q*-regime. The ASAXS measurements reveal a superior degree of recovery when compared to SAXS at higher volume fractions.

In order to make optimal use of these simulations, with respect to an appropriate evaluation of our quantitative RI-analysis, we adopt the following point of view. If both samples would lie on the phase boundary for Pb-PA in 0.1 M NaNO_3_, given in [24] as:

[Pb^2+^]_c_ ~0.28[COO^−^]_c_

with [COO^−^]_c_ and [Pb^2+^]_c_ the concentration of NaPA and Pb^2+^ in moles per liter at the threshold, the degree of decoration of both samples would be [Pb^2+^]/[COO^−^] ~0.3. According to the selected values of [COO^−^] and [Pb^2+^] of the two samples (Table 1) this is not possible. However, we can still assume as a limiting case that all Pb^2+^ ions available in solution are bound by COO^−^-residues of the NaPA chains and that those bound Pb^2+^ ions are all trapped in collapsed pearl-like confinements. This assumption leads us to assign a degree of decoration of [Pb^2+^]/[COO^−^] ~0.21 (FRM01) and [Pb^2+^]/[COO^−^] ~0.24 (FRM02), and to conclude that the present RI-analysis, which is based on an integral according to Equation (8) up to *q* = 2 nm^−1^, has recovered just 11% and 14% of those Pb^2+^ ions which presumably decorate the NaPA chains and are, thereby, condensed to confinements. Entering into Figure 5b with these percentages (the two horizontal lines), it can be realized that such a discrepancy is only compatible with a confinement size *D* of 2 < *D* < 6.4 nm filled with a number of Pb^2+^ ions representing volume fractions of Pb^2+^ ions in the confinement between 10 and 25%. The regime of compatible confinement sizes is represented by the grey area in Figure 5b. This is in excellent agreement with the results deduced from the FJC-PN model which results in confinements larger than 1 nm.

If, unlike to the just-adopted point of view, not all Pb^2+^ ions in solution are bound by chains, *i.e.*, in case of a significant portion of Pb^2+^ ions moving freely in solution and, thus, scattering only incoherently the number of Pb^2+^ ions recovered as being bound to chains by the restricted integral up to 2 nm^−1^ must be larger than 11%–14%, which in Figure 5b can only be accommodated by a volume fraction of Pb^2+^ ions in the confinement larger than ~20%.

In order to shed more light on the consequences of the latter, we present a gedankenexperiment. Let us assume that only 50% of the Pb^2+^ ions are in the collapsed subdomains while the other 50% are free in solution and not contributing to the small-angle scattering of the collapsed subdomains. Given, now, that 11% and 14% of the ASAXS active species are recovered by RI analysis, 22% and 28% of the counterions have to be in the condensed phase because, per definition, 50% of the Pb^2+^ ions cannot be detected by ASAXS. Entering with these numbers Figure 5b (horizontal lines now at 22% and 28%) provides volume fractions between 25% and 50% for the confinements with sizes between 2 and 6.5 nm. This seems to mark an upper limit of the volume fractions for Pb^2+^, because along with the filling by Pb^2+^ ions additional volume has to be allocated to the hosting polymer segments and, most likely, some volume is also occupied by residual solvent molecules.

## 5. Conclusions

Diluted solutions of anionic polyacrylates decorated with specifically interacting Pb^2+^ cations have been analyzed by small-angle neutron scattering and anomalous small-angle X-ray scattering experiments in the energy range of the L_III_-absorption edge of lead. From the SANS and SAXS measurements, structural information of the organic confinements, which form via condensation of specifically-interacting Pb^2+^ ions was deduced. This information suggests that the polyacrylate chains host the complex bound Pb^2+^ ions in collapsed subdomains with a size of 2.6 < *R* < 3.4 nm where R is the radius of these collapsed pearl-like confinements. Moreover, from the resonant invariant, the amount of Pb^2+^ ions located in these pearls was determined. Comparison with XANES measurements revealed that the deduced concentration values are smaller by a factor 1/7 to 1/9. It is this point, where significant progress could be achieved with Monte Carlo simulations, which provided an instructive picture of the limitations of the quantitative amount of ASAXS active entities deduced from an integration of the Kratky plot. The simulations revealed estimations of the accessible fraction of the true amount of bound ions, as it depends on the integration limit, the size of the confinements and the degree of filling of the confinements with ASAXS active entities. Applied to our system, the Monte Carlo simulations demonstrate, that if a RI is based on an integral limited to 2 nm^−1^ and if ASAXS active species are accumulated in confinements of a few nanometers in size, the degree which can be recovered from those accumulated ASAXS active species can show strong variation between a few percent up to 70% depending on the degree of filling. From this findings it was concluded, that the filling degree of bound entities must be between 10% and 25% in order to achieve compatible results when comparing the concentration values obtained from RI-analysis and from XANES. If a significant portion of counterions would not have bound to the polyacrylate chains, our Monte Carlo simulations would have suggested a filling degree in the condensed domains larger than 20%.

## Figures and Tables

**Table 1:** The concentrations of polyacrylate monomers and of Pbcounterions of the samples investigated in the present study: [Pb^2+^]_Prep_ known from preparation, [Pb^2+^]_XANES_ deduced from XANES measurements, and [Pb^2+^]_ASAXS_ deduced from RI-analysis. Δμd was taken from XANES measurements (see below).

Sample	Polyacrylate [g/L] [g/mmol] [10^17^cm^−3^]	[Pb^2+^]_Prep_ [mmol/L]	[Pb^2+^]_Prep_ 10^17^cm^−3^	Δμd	[Pb^2+^]_XANES_ 10^17^cm^−3^	[Pb^2+^]_ASAXS_ 10^17^cm^−3^	[Pb^2+^]_ASAXS_ /[Pb^2+^]_XANES_
FRM01	0.7575 8.06 48	1.65	9.9	0.0038(5)	8.5(9)	0.97(8)	0.11
FRM02	0.6565 6.98 42	1.65	9.9	0.0039(5)	8.7(9)	1.20(6)	0.14

**Table 2:** The anomalous dispersion corrections of Pb at the three energies of the ASAXS experiment based on the calculations of Cromer and Liberman [35,36].

E [eV]	f’	f’’
12,650	−9.982	4.134
13,000	−14.602	3.956
13,035	−22.666	6.002

## Supplementary Materials

Supplementary materials can be found at www.mdpi.com/2073-4360/8/3/85/s1.

## References

[1] Nagasawa M. (1988). Studies in Polymer Science.

[2] Huber K. (1993). Calcium-induced shrinking of polyacrylate chains in aqueous solution. J. Phys. Chem..

[3] Michaeli I. (1960). Ion binding and the formation of insoluble polymethacrylic salts. J. Polym. Sci..

[4] Kantor Y., Kardar M. (1994). Excess charge in polyampholytes. Europhys. Lett..

[5] Dobrynin A.V., Rubinstein M., Obukhov S.P. (1996). Cascade of transitions of polyelectrolytes in poor solvents. Macromolecules.

[6] Solis F.J., Olvera de la Cruz M. (1998). Variational approach to necklace formation in polyelectrolytes. Macromolecules.

[7] Micka U., Holm C., Kremer K. (1999). Strongly charged, flexible polyelectrolytes in poor solvents: Molecular dynamics simulations. Langmuir.

[8] Chodanowski P., Stoll S. (1999). Monte Carlo simulations of hydrophobic polyelectrolytes: Evidence of complex configurational transitions. J. Chem. Phys..

[9] Lyulin A.V., Dünweg B., Borisov O.V., Darinskii A.A. (1999). Computer simulation studies of a single polyelectrolyte chain in poor solvent. Macromolecules.

[10] Limbach H.J., Holm C. (2002). End effects of strongly charged polyelectrolytes: A molecular dynamics study. J. Phys. Chem. B.

[11] Aseyev V.O., Klenin S.I., Tenhu H., Grillo I., Geissler E. (2001). Neutron scattering studies of the structure of a polyelectrolyte globule in a water-acetone mixture. Macromolecules.

[12] Li M.-J., Green M.M., Morawetz H. (2002). NMR spectra of polyelectrolytes in poor solvents are consistent with the pearl necklace model of the chain molecules. Macromolecules.

[13] Minko S., Kiriy A., Gorodyska G., Stamm M. (2002). Mineralization of single flexible polyelectrolyte molecules. J. Am. Chem. Soc..

[14] Schweins R., Huber K. (2001). Collapse of sodium polyacrylate chains in calcium salt solutions. Eur. Phys. J. E.

[15] Goerigk G., Schweins R., Huber K., Ballauff M. (2004). The distribution of Sr^2+^ counterions around polyacrylate chains analyzed by anomalous small-angle X-ray scattering. Europhys. Lett..

[16] Stuhrmann H.B. (1985). Resonance scattering in macromolecular structure research. Adv. Polym. Sci..

[17] De Robilliard Q., Guo X., Dingenouts N., Ballauff M., Goerigk G. (2001). Application of anomalous small-angle X-ray scattering to spherical polyelectrolyte brushes. Macromol. Symp..

[18] Guilleaume B., Ballauff M., Goerigk G., Wittemann M., Rehahn M. (2001). Correlation of counterions with rodlike macroions as assessed by anomalous small-angle X-ray scattering. Colloid Polym. Sci..

[19] Guilleaume B., Blaul J., Ballauff M., Wittemann M., Rehahn M., Goerigk G. (2002). The distribution of counterions around synthetic rod-like polyelectrolytes in solution. Eur. Phys. J..

[20] Sabbagh I., Delsanti M., Lesieur P. (1999). Ionic distribution and polymer conformation, near phase separation, in sodium polyacrylate/divalent cations mixtures: Small angle X-ray and neutron scattering. Eur. Phys. J. B.

[21] Das R., Mills T.T., Kwok L.W., Maskel G.S., Millet L.S., Doniach S., Finkelstein K.D., Herschlag D., Pollack L. (2003). Counterion distribution around DNA Probed by solution X-ray scattering. Phys. Rev. Lett..

[22] Schweins R., Goerigk G., Huber K. (2006). Shrinking of anionic polyacrylate coils induced by Ca^2+^, Sr^2+^ and Ba^2+^: A combined light scattering and ASAXS study. Eur. Phys. J. E.

[23] Goerigk G., Huber K., Schweins R. (2007). Probing the extent of the Sr^2+^ ion condensation to anionic polyacrylate coils: A quantitative anomalous small-angle x-ray scattering study. J. Chem. Phys..

[24] Lages S., Goerigk G., Huber K. (2013). SAXS and ASAXS on dilute sodium polyacrylate chains decorated with lead ions. Macromolecules.

[25] Michels R., Goerigk G., Vainio U., Gummel J., Huber K. (2014). Coaggregation of two anionic azo dyestuffs: A combined static light scattering and small-angle X-ray scattering study. J. Phys. Chem. B.

[26] Goerigk G., Mattern N. (2010). Spinodal decomposition in Ni-Nb-Y metallic glasses analyzed by quantitative anomalous small-angle X-ray scattering. J. Phys..

[27] Goerigk G., Huber K., Mattern N., Williamson D.L. (2012). Quantitative anomalous small-angle X-ray scattering—The determination of chemical concentrations in nano-scaled phases. Eur. Phys. J. Spec. Top..

[28] Goerigk G. (2013). The Solution of the Eigenvector problem in synchrotron radiation based anomalous small-angle X-ray scattering. ALAMT.

[29] Guinier A., Fournet G. (1955). Small-Angle Scattering of X-Rays.

[30] Glatter O., Kratky O. (1982). Small-Angle X-Ray Scattering.

[31] Goerigk G., Mattern N. (2009). Critical scattering of Ni–Nb–Y metallic glasses probed by quantitative anomalous small-angle X-ray scattering. Acta Mater..

[32] Goerigk G., Williamson D.L. (2006). Temperature induced differences in the nanostructure of hot-wire deposited silicon-germanium alloys analyzed by anomalous small-angle X-ray scattering. J. Appl. Phys..

[33] Goerigk G. (2006). Electronic and Computer Upgrade at ASAXS Beamline JUSIFA.

[34] Haubold H.-G., Gruenhagen K., Wagener M., Jungbluth H., Heer H., Pfeil A., Rongen H., Brandenburg G., Moeller R., Matzerath J. (1989). JUSIFA—A new user-dedicated ASAXS beamline for materials science. Rev. Sci. Instrum..

[35] Cromer D.T., Liberman D. (1970). Relativistic calculation of anomalous scattering factors for X rays. J. Chem. Phys..

[36] Cromer D.T., Liberman D. (1981). Anomalous dispersion calculations near to and on the long-wavelength side of an absorption edge. Acta Cryst..

[37] Radulescu A., Pipich V., Frielinghaus H., Appavou M.S. (2012). KWS-2, the high intensity/wide Q-range small-angle neutron diffractometer for soft-matter and biology at FRM II. J. Phys. Conf. Ser..

