# Supplementary material for: Systematic Limitations in Concentration Analysis via Anomalous Small-Angle X-ray Scattering in the Small Structure Limit
Source: Polymers (Basel). 2016 Mar 16;8(3):85. doi: 10.3390/polym8030085 (PMC6432554; doi:10.3390/polym8030085)
Supplement: Supplementary file 1 [file polymers-08-00085-s001.pdf]

Supplementary Materials: Systematic Limitations in Concentration Analysis via Anomalous Small-Angle X-ray Scattering in the Small Structure Limit

Günter Goerigk, Sebastian Lages and Klaus Huber

The Calculation of the Resonant Invariant of Point-like Scattering Centers in Confinements

The spatial distribution of point-like scattering centers located at $\vec{r}_i$ is:

$$v(\vec{r}) = \sum_{i=1}^{N} \delta(\vec{r} - \vec{r}_i) \tag{1}$$

From this the form factor *in vacuo* can be deduced:

$$\begin{aligned} |A(\vec{q})|^2 &= \iint_{R^3} v(\vec{r}) v(\vec{r}') e^{i\vec{q}(\vec{r}-\vec{r}')} d^3r d^3r' \\ &= \iint_{R^3} \sum_{i,j=1}^{N} \delta(\vec{r} - \vec{r}_i)\delta(\vec{r}' - \vec{r}_j) e^{i\vec{q}(\vec{r}-\vec{r}')} d^3r d^3r' = \sum_{i,j=1}^{N} e^{i\vec{q}(\vec{r}_i - \vec{r}_j)} \end{aligned} \tag{2}$$

The invariant of the point-like scatteres writes:

$$\begin{aligned} Q &= \int_{Q^3} |A(\vec{q})|^2 d^3q = \int_{Q^3} \sum_{i,j=1}^{N} e^{i\vec{q}(\vec{r}_i - \vec{r}_j)} d^3q = \sum_{i,j=1}^{N} \int_0^{2\pi}\int_0^{\pi}\int_0^{\infty} e^{iq|\vec{r}_i - \vec{r}_j|\cos(\vartheta)} \sin(\vartheta) d\vartheta d\phi q^2 dq \\ &= 2\pi \sum_{i,j=1}^{N} \int_0^{\pi}\int_0^{\infty} e^{iq|\vec{r}_i - \vec{r}_j|\cos(\vartheta)} (-1) d\cos(\vartheta) q^2 dq \\ &= 2\pi \sum_{i,j=1}^{N} \int_1^{-1}\int_0^{\infty} e^{iq|\vec{r}_i - \vec{r}_j|x} (-1) dx q^2 dq \\ &= 2\pi \sum_{i,j=1}^{N} \int_{-1}^{+1}\int_0^{\infty} e^{iq|\vec{r}_i - \vec{r}_j|x} dx q^2 dq \\ &= 2\pi \sum_{i,j=1}^{N} \int_0^{\infty} \left. \frac{e^{iq|\vec{r}_i - \vec{r}_j|x}}{iq|\vec{r}_i - \vec{r}_j|} \right|_{-1}^{+1} q^2 dq \\ &= 2\pi \sum_{i,j=1}^{N} \int_0^{\infty} \frac{2i \sin(q|\vec{r}_i - \vec{r}_j|)}{iq|\vec{r}_i - \vec{r}_j|} q^2 dq \\ &= 4\pi \sum_{i,j=1}^{N} \int_0^{\infty} \frac{\sin(q|\vec{r}_i - \vec{r}_j|)}{q|\vec{r}_i - \vec{r}_j|} q^2 dq \\ &= 4\pi \left\{ N\int_0^{\infty} q^2 dq + 2 \sum_{i=1, j>i}^{N} \frac{1}{|\vec{r}_i - \vec{r}_j|} \int_0^{\infty} \sin(q|\vec{r}_i - \vec{r}_j|) q dq \right\} \end{aligned} \tag{3}$$

The invariant is composed of the incoherent scattering, which originates from the single point scatters (first term) and the coherent scattering from the overall assembly (second term). Since the elastic scattering of photons is limited by the maximum possible scattering angle, 2θ = 180°, and by the wavelength of the photons of the experiment under consideration setting q_{max}, the integration in the last line of Equation (3) can be restricted to q_{max}. Beyond this value no (elastic) scattering can

appear in the reciprocal space. When performing ASAXS measurements in the energy range of the L_{III}-absorption edge of Pb (13,035 eV) the related wavelength is 0.091 nm giving:

$$q_{\max} = 136 \text{ nm}^{-1}$$

Thus, the integration over the entire reciprocal space of a given experiment can now be restricted in Equation (3) up to $q_{\max}$ giving:

$$\begin{aligned}
&= 4\pi\left\{N\frac{1}{3}q^3\Big|_0^{q_{\max}} + 2\sum_{i=1,j>i}^{N}\frac{1}{|\vec{r}_i-\vec{r}_j|}\left[\frac{-\cos(q|\vec{r}_i-\vec{r}_j|)}{|\vec{r}_i-\vec{r}_j|}q\Big|_0^{q_{\max}} - \int_0^{q_{\max}}\frac{-\cos(q|\vec{r}_i-\vec{r}_j|)}{|\vec{r}_i-\vec{r}_j|}dq\right]\right\} \\
&= 4\pi\left\{N\frac{1}{3}q_{\max}^3 + 2\sum_{i=1,j>i}^{N}\frac{1}{|\vec{r}_i-\vec{r}_j|^2}\left[\frac{\sin(q|\vec{r}_i-\vec{r}_j|)}{|\vec{r}_i-\vec{r}_j|}\Big|_0^{q_{\max}} - q_{\max}\cos(q_{\max}|\vec{r}_i-\vec{r}_j|)\right]\right\} \\
&= 4\pi\left\{N\frac{1}{3}q_{\max}^3 + 2\sum_{i=1,j>i}^{N}\frac{1}{|\vec{r}_i-\vec{r}_j|^3}\left[\sin(q_{\max}|\vec{r}_i-\vec{r}_j|) - q_{\max}|\vec{r}_i-\vec{r}_j|\cos(q_{\max}|\vec{r}_i-\vec{r}_j|)\right]\right\} \qquad (4) \\
&= 4\pi\left\{N\frac{1}{3}q_{\max}^3 + \frac{2}{3}\sum_{i=1,j>i}^{N}\frac{q_{\max}^3}{q_{\max}^3|\vec{r}_i-\vec{r}_j|^3}3\left[\sin(q_{\max}|\vec{r}_i-\vec{r}_j|) - q_{\max}|\vec{r}_i-\vec{r}_j|\cos(q_{\max}|\vec{r}_i-\vec{r}_j|)\right]\right\} \\
&= \frac{4\pi}{3}q_{\max}^3\left\{N + 2\sum_{i=1,j>i}^{N}3\frac{\left[\sin(q_{\max}|\vec{r}_i-\vec{r}_j|) - q_{\max}|\vec{r}_i-\vec{r}_j|\cos(q_{\max}|\vec{r}_i-\vec{r}_j|)\right]}{q_{\max}^3|\vec{r}_i-\vec{r}_j|^3}\right\}
\end{aligned}$$

Now we turn to the description of N point-like scatterers embedded in an isolated confinement of averaged scattering center density $\bar{v}$. Only inhomogeneities which differ in scattering center density with respect to the average scattering center density contribute to the small-angle scattering (Porod) [30]. The difference of the scattering center density from its spatially-averaged value is, thus, written as:

$$\begin{aligned}
&\upsilon(\vec{r}) = f(\vec{r})\Big(\sum_{i=1}^{N}\delta(\vec{r}-\vec{r}_i) - \bar{v}\Big) \\
&f(\vec{r}) = 1; \qquad \vec{r} \in V_{\mathrm{P}} \qquad (5) \\
&f(\vec{r}) = 0; \qquad \vec{r} \notin V_{\mathrm{P}}
\end{aligned}$$

The function $f(\vec{r})$ represents the shape of the confinement with the volume V_{P}. Integration of the as-defined scattering length density yields zero as requested by Porod's theory:

$$\begin{aligned}
\int_{R^3}\upsilon(\vec{r})d^3r &= \int_{R^3} f(\vec{r})\Big(\sum_{i=1}^{N}\delta(\vec{r}-\vec{r}_i) - \bar{v}\Big)d^3r = \int_{V_P}\Big(\sum_{i=1}^{N}\delta(\vec{r}-\vec{r}_i) - \bar{v}\Big)d^3r \\
&= \sum_{i=1}^{N}\int_{V_P}\delta(\vec{r}-\vec{r}_i)d^3r - \bar{v}\int_{V_P}d^3r \\
&= N - \bar{v}\int_{V_P}d^3r \qquad (6) \\
&= N - \bar{v}V_P = N - \frac{N}{V_{\mathrm{P}}}V_{\mathrm{P}} \\
&= N - N = 0
\end{aligned}$$

Since $f(\vec{r})$ differs from zero only in the interior of the confinement the integration limits of the third integral in the first line of Equation (6) shrink to V_P. The same result is obtained for the general

case of many confinements and/or additional ions not located inside a confinement. This case is not treated here because our Monte Carlo simulations have been restricted to only one confinement.

If we use the difference of the scattering centre density $\upsilon(\vec{r})$ from its average, the double integral in Equation (2) reveals for the form factor:

$$
\begin{aligned}
|A(\vec{q})|^2 &= \iint_{R^3} \upsilon(\vec{r})\upsilon(\vec{r}')e^{i\vec{q}(\vec{r}-\vec{r}')}d^3r d^3r' \\
&= \iint_{R^3} \sum_{i,j=1}^{N} f(\vec{r})(\delta(\vec{r}-\vec{r}_i)-\bar{v})f(\vec{r}')(\delta(\vec{r}'-\vec{r}_j)-\bar{v})e^{i\vec{q}(\vec{r}-\vec{r}')}d^3r d^3r' \\
&= \iint_{R^3} \sum_{i,j=1}^{N} f(\vec{r})\delta(\vec{r}-\vec{r}_i)f(\vec{r}')\delta(\vec{r}'-\vec{r}_j)e^{i\vec{q}(\vec{r}-\vec{r}')}d^3r d^3r' \\
&- \bar{v}\iint_{R^3} \sum_{i=1}^{N} f(\vec{r})f(\vec{r}')\delta(\vec{r}-\vec{r}_i)e^{i\vec{q}(\vec{r}-\vec{r}')}d^3r d^3r' \\
&- \bar{v}\iint_{R^3} \sum_{j=1}^{N} f(\vec{r}')f(\vec{r})\delta(\vec{r}'-\vec{r}_j)e^{i\vec{q}(\vec{r}-\vec{r}')}d^3r d^3r' \\
&+ \bar{v}^2\iint_{R^3} f(\vec{r})f(\vec{r}')e^{i\vec{q}(\vec{r}-\vec{r}')}d^3r d^3r'
\end{aligned}
\tag{7}
$$

Due to the shape function of the confinement the integration limits shrink to the volume V_P of the confinement:

$$
\begin{aligned}
&= \iint_{V_p} \sum_{i,j=1}^{N} \delta(\vec{r}-\vec{r}_i)\delta(\vec{r}'-\vec{r}_j)e^{i\vec{q}(\vec{r}-\vec{r}')}d^3r d^3r' \\
&- \bar{v}\iint_{V_p} \sum_{i=1}^{N} \delta(\vec{r}-\vec{r}_i)e^{i\vec{q}(\vec{r}-\vec{r}')}d^3r d^3r' \\
&- \bar{v}\iint_{V_p} \sum_{j=1}^{N} \delta(\vec{r}'-\vec{r}_j)e^{i\vec{q}(\vec{r}-\vec{r}')}d^3r d^3r' \\
&+ \bar{v}^2\iint_{V_p} e^{i\vec{q}(\vec{r}-\vec{r}')}d^3r d^3r'
\end{aligned}
\tag{8}
$$

$$
= \sum_{i,j=1}^{N} e^{i\vec{q}(\vec{r}_i-\vec{r}_j)} - \bar{v}\int_{V_p}\sum_{i=1}^{N} e^{i\vec{q}(\vec{r}_i-\vec{r}')}d^3r' - \bar{v}\int_{V_p}\sum_{j=1}^{N} e^{i\vec{q}(\vec{r}-\vec{r}_j)}d^3r + \bar{v}^2\iint_{V_p} e^{i\vec{q}(\vec{r}-\vec{r}')}d^3r d^3r'
$$

The invariant is calculated from the form factor via integration over the entire reciprocal space of $0 \leq q < \infty$:

$$
\begin{aligned}
Q &= \int_{Q^3} \left|A(\vec{q})\right|^2 d^3q \\
&= \int_{Q^3} \sum_{i,j=1}^{N} e^{i\vec{q}(\vec{r}_i-\vec{r}_j)} d^3q - \int_{Q^3} \bar{v} \int_{V_p} \sum_{i=1}^{N} e^{i\vec{q}(\vec{r}_i-\vec{r}')} d^3r' d^3q \\
&- \int_{Q^3} \bar{v} \int_{V_p} \sum_{j=1}^{N} e^{i\vec{q}(\vec{r}-\vec{r}_j)} d^3r d^3q + \int_{Q^3} \bar{v}^2 \iint_{V_p} e^{i\vec{q}(\vec{r}-\vec{r}')} d^3r d^3r' d^3q \\
&= \int_{Q^3} \sum_{i,j=1}^{N} e^{i\vec{q}(\vec{r}_i-\vec{r}_j)} d^3q - \bar{v} \int_{V_p} \sum_{i=1}^{N} (2\pi)^3 \delta(\vec{r}_i - \vec{r}') d^3r' \\
&- \bar{v} \int_{V_p} \sum_{j=1}^{N} (2\pi)^3 \delta(\vec{r} - \vec{r}_j) d^3r + \bar{v}^2 \iint_{V_p} (2\pi)^3 \delta(\vec{r} - \vec{r}') d^3r d^3r' \\
&= \int_{Q^3} \sum_{i,j=1}^{N} e^{i\vec{q}(\vec{r}_i-\vec{r}_j)} d^3q - (2\pi)^3 \bar{v} \sum_{i=1}^{N} 1 - (2\pi)^3 \bar{v} \sum_{j=1}^{N} 1 + (2\pi)^3 \bar{v}^2 \iint_{V_p} \delta(\vec{r} - \vec{r}') d^3r d^3r' \\
&= \int_{Q^3} \sum_{i,j=1}^{N} e^{i\vec{q}(\vec{r}_i-\vec{r}_j)} d^3q - (2\pi)^3 N\bar{v} - (2\pi)^3 N\bar{v} + (2\pi)^3 \bar{v}^2 \int_{V_p} d^3r
\end{aligned}
\tag{9}
$$

The last integral is restricted to the sample volume which, in the present case, is the confinement defined by $f(\vec{r})$, thereby rendering the sample volume to the confinement volume:

$$
\begin{aligned}
& \int_{Q^3} \sum_{i,j=1}^{N} e^{i\vec{q}(\vec{r}_i-\vec{r}_j)} d^3q - (2\pi)^3 N\bar{v} - (2\pi)^3 N\bar{v} + (2\pi)^3 \bar{v}^2 V_{\mathrm{p}} \\
&= \int_{Q^3} \sum_{i,j=1}^{N} e^{i\vec{q}(\vec{r}_i-\vec{r}_j)} d^3q - 2(2\pi)^3 \bar{v}^2 V_P + (2\pi)^3 \bar{v}^2 V_{\mathrm{P}} \\
&= \int_{Q^3} \sum_{i,j=1}^{N} e^{i\vec{q}(\vec{r}_i-\vec{r}_j)} d^3q - (2\pi)^3 \bar{v}^2 V_{\mathrm{P}} \\
&= \int_{Q^3} \sum_{i,j=1}^{N} e^{i\vec{q}(\vec{r}_i-\vec{r}_j)} d^3q - (2\pi)^3 N\bar{v}
\end{aligned}
\tag{10}
$$

with $\bar{v} = \dfrac{N}{V_{\mathrm{P}}}$. In the last line of Equation (10) we identify the integral of the first line in Equation (3), which has already been explicitly calculated in Equations (3) and (4). Thus, the final result is obtained via replacing the integral in the last line of Equation (10) by the result taken from the last line in Equation (4). We rename the invariant to Q_{max} in order to emphasize the crucial role of the integration performed over the entire reciprocal space to q_{max}:

$$
\begin{aligned}
Q_{\mathrm{max}} &= \frac{4\pi}{3} q_{\mathrm{max}}^3 \left\{ N + 2 \sum_{i=1, j>i}^{N} 3 \frac{\left[\sin(q_{\mathrm{max}}|\vec{r}_i - \vec{r}_j|) - q_{\mathrm{max}}|\vec{r}_i - \vec{r}_j| \cos(q_{\mathrm{max}}|\vec{r}_i - \vec{r}_j|)\right]}{q_{\mathrm{max}}^3 |\vec{r}_i - \vec{r}_j|^3} \right\} \\
&- (2\pi)^3 N\bar{v}
\end{aligned}
\tag{11}
$$

The final result for the first term in Equation (11) gives reliable (exact) values only when the integration is extended to q_{max}. If the integration terminates at smaller q-values the oscillating second term in the bracket can produce negative values (which are not compensated by related positive values occurring at larger *q*-values), thereby producing values for Q which are far too low or *vice versa*. The latter is explicitly depicted in Figure 4b of the main text. Thus, only Q_{max} gives a meaningful value which can be further processed in order to determine the concentration of the scattering centers. The situation is different in the small-angle scattering regime because here, due to constructive interference of the scattering amplitudes, the scattering intensity is preferentially accumulated in the low q-range and Porod's theory applies.

© 2016 by the authors; licensee MDPI, Basel, Switzerland. This article is an open access article distributed under the terms and conditions of the Creative Commons by Attribution (CC-BY) license (http://creativecommons.org/licenses/by/4.0/).
